# Liver-specific insulin receptor isoform A expression enhances hepatic glucose uptake and ameliorates liver steatosis in a mouse model of diet-induced obesity

**DOI:** 10.1242/dmm.036186

**Published:** 2019-02-07

**Authors:** Andrea Raposo Lopez-Pastor, Almudena Gomez-Hernandez, Sabela Diaz-Castroverde, Gloria Gonzalez-Aseguinolaza, Agueda Gonzalez-Rodriguez, Gema Garcia, Silvia Fernandez, Oscar Escribano, Manuel Benito

**Affiliations:** 1 Department of Biochemistry and Molecular Biology, School of Pharmacy, Complutense University of Madrid, 28040 Madrid, Spain; 2 CIBER of Diabetes and Related Diseases (CIBERDEM), Health Institute Carlos III (ISCIII), 28029 Madrid, Spain; 3 Division of Hepatology and Gene Therapy, Center for Applied Medical Research, University of Navarra, 31008 Pamplona, Spain; 4 Liver Research Unit, Hospital Universitario Santa Cristina, Instituto de Investigación Sanitaria Princesa, Amadeo Vives 2, 28009 Madrid, Spain; 5 CIBER of Hepatic and Digestive Diseases (CIBERehd), 28029 Madrid, Spain

**Keywords:** Glucose metabolism, Insulin receptor isoforms, Adeno-associated viruses, Gene therapy, Non-alcoholic fatty liver disease, Insulin resistance

## Abstract

Among the main complications associated with obesity are insulin resistance and altered glucose and lipid metabolism within the liver. It has previously been described that insulin receptor isoform A (IRA) favors glucose uptake and glycogen storage in hepatocytes compared with isoform B (IRB), improving glucose homeostasis in mice lacking liver insulin receptor. Thus, we hypothesized that IRA could also improve glucose and lipid metabolism in a mouse model of high-fat-diet-induced obesity. We addressed the role of insulin receptor isoforms in glucose and lipid metabolism *in vivo*. We expressed IRA or IRB specifically in the liver by using adeno-associated viruses (AAVs) in a mouse model of diet-induced insulin resistance and obesity. IRA, but not IRB, expression induced increased glucose uptake in the liver and muscle, improving insulin tolerance. Regarding lipid metabolism, we found that AAV-mediated IRA expression also ameliorated hepatic steatosis by decreasing the expression of *Fasn*, *Pgc1a*, *Acaca* and *Dgat2* and increasing *Scd-1* expression. Taken together, our results further unravel the role of insulin receptor isoforms in hepatic glucose and lipid metabolism in an insulin-resistant scenario. Our data strongly suggest that IRA is more efficient than IRB at favoring hepatic glucose uptake, improving insulin tolerance and ameliorating hepatic steatosis. Therefore, we conclude that a gene therapy approach for hepatic IRA expression could be a safe and promising tool for the regulation of hepatic glucose consumption and lipid metabolism, two key processes in the development of non-alcoholic fatty liver disease associated with obesity.

This article has an associated First Person interview with the first author of the paper.

## Abstract (precis)

**Summary:** Adeno-associated-virus-mediated gene therapy for insulin receptor isoform A expression in the liver improves glucose disposal and alleviates lipid accumulation in wild-type mice under a high-fat diet.

## INTRODUCTION

Obesity is widely considered the epidemic of the 21st century (NCD Risk Factor Collaboration, 2016; Gonzalez-Muniesa et al., 2017; Reilly and Saltiel, 2017), and is characterized by a huge body weight for height, with increased accumulation of adipose tissue. It is usually accompanied by mild, chronic and systemic inflam­mation. Moreover, obesity is highly associated with the development of type 2 diabetes mellitus (T2DM), non-alcoholic fatty liver disease (NAFLD), cardiovascular diseases (CVDs) and other adverse pathological condi­tions (Williams et al., 2015).

T2DM development is characterized by both insulin resistance and impaired insulin secretion, with insulin resistance the most important feature in pre-diabetic states (Reilly and Saltiel, 2017). Although insulin resistance affects many tissues, including muscle and adipose tissue, the liver – involved in the regulation of blood glucose homeostasis by glucose uptake and storage in the postprandial state – is one of the main organs affected (von Wilamowitz-Moellendorff et al., 2013). Therefore, hepatic insulin resistance becomes a hallmark of T2DM development (Cook et al., 2015), and precise regulation of glucose homeostasis is essential for proper diabetes management (Callejas et al., 2013).

The insulin receptor (IR) is a tyrosine kinase receptor with a major role in glucose metabolism (Benyoucef et al., 2007; Ward and Lawrence, 2009). In mammals, alternative splicing gives rise to two isoforms: IRA and IRB, the latter containing 12 additional amino acids encoded by exon 11 (Whittaker et al., 2002). This sequence is immediately located downstream of the ligand-binding domain but does not affect insulin-binding affinity, although it does affect the binding affinity of IGF proteins; the lack of exon 11 enables an increased affinity mainly for IGF-II but also for IGF-I (Menting et al., 2013; Whittaker et al., 2002). IRA is mainly expressed during embryogenesis and perinatal life when signals for IGF-II occur (Frasca et al., 1999). However, IRB is predominantly expressed in adult tissues, including the liver, where it triggers the metabolic effects of insulin (Malaguarnera and Belfiore, 2011).

Although hepatic glucose uptake is insulin independent, previous studies performed in cultured hepatocytes demonstrated that IRA plays a direct role in favoring glucose uptake through its specific association with endogenous GLUT1 and GLUT2 (Nevado et al., 2006). Therefore, differences in glucose uptake could be explained by the presence/absence of IR isoforms. In this sense, our group demonstrated *in vivo* that AAV-mediated IRA expression, specifically in the liver, improved glucose intolerance in a mouse model of hepatic insulin resistance (Diaz-Castroverde et al., 2016b). Moreover, we also demonstrated that this improvement in glucose tolerance was accompanied by an increased hepatic glycogen storage (Diaz-Castroverde et al., 2016a), suggesting that hepatic expression of IRA could serve as a glucose uptake facilitator in insulin-resistant states.

With this background, and to further validate AAV-mediated hepatic IRA expression as a potential gene therapy tool for decreasing hyperglycemia in insulin-resistant states, we hypothesized that this gene therapy approach could also work in a mouse model of diet-induced obesity. In the current study, we expressed IRA or IRB specifically in the liver of high-fat diet (HFD)-fed wild-type mice and demonstrate a differential role of IR isoforms in glucose and lipid metabolism.

## RESULTS

**Fig. 1.**
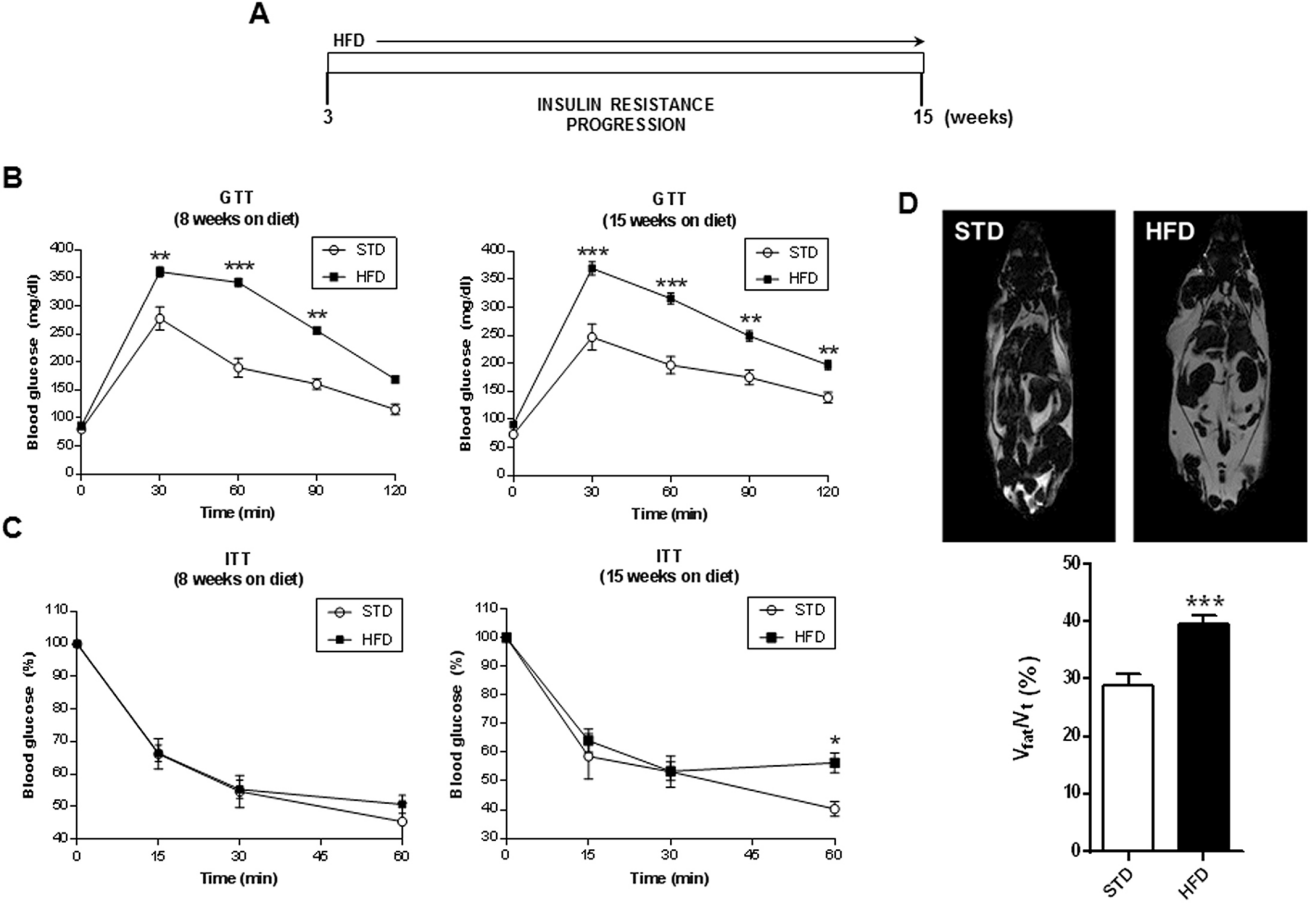
**Characterization of the mouse model of diet-induced insulin resistance and obesity.** (A) Scheme of HFD-induced insulin resistance progression. (B) Glucose tolerance test (GTT) in STD (*n*=5) and HFD (*n*=21) mice at 8 (left) or 15 (right) weeks on the diet. (C) Insulin tolerance test (ITT) in STD (*n*=5) and HFD (*n*=21) mice at 8 (left) or 15 (right) weeks on the diet. (D) Representative nuclear magnetic resonance (NMR) images of STD (top left) and HFD (top right) mice; quantification of NMR images of STD (*n*=5) and HFD (*n*=9) groups prior to AAV administration (bottom). Results are expressed as mean±s.e.m. Statistical significance was assessed by one-way ANOVA with Bonferroni post test in B and C, and by two-tailed unpaired Student's *t*-test in D, comparing HFD mice with the STD group (*P<0.05, **P<0.01 and ***P<0.001).

Previous studies carried out in our laboratory on inducible liver IR knockout (iLIRKO) mice demonstrated that AAV-mediated hepatic expression of IRA triggered an improvement in the diabetic phenotype (Diaz-Castroverde et al., 2016b). Given that obesity and T2DM are closely related, we wanted to expand these studies to a murine model of HFD-induced insulin resistance, and to explore whether IRA could also work as a therapeutic tool in these conditions. After weaning, animals were organized into two groups, one fed a standard diet (STD) and the other fed a HFD. Initially, the animals were analyzed following the scheme showed in Fig. 1A in order to check the development of insulin resistance. Glucose tolerance tests (GTTs) and insulin tolerance tests (ITTs) were performed 8 and 15 weeks after weaning (Fig. 1B,C). Our results show that 8 and 15 weeks after HFD administration, mice developed an overt and maintained glucose intolerance. Moreover, insulin resistance was observed after 15 weeks of HFD. We also performed magnetic resonance imaging experiments to evaluate fat accumulation and observed a very significant increase in the ratio of fat volume/total volume in HFD mice compared with the STD group (Fig. 1D).

**Fig. 2.**
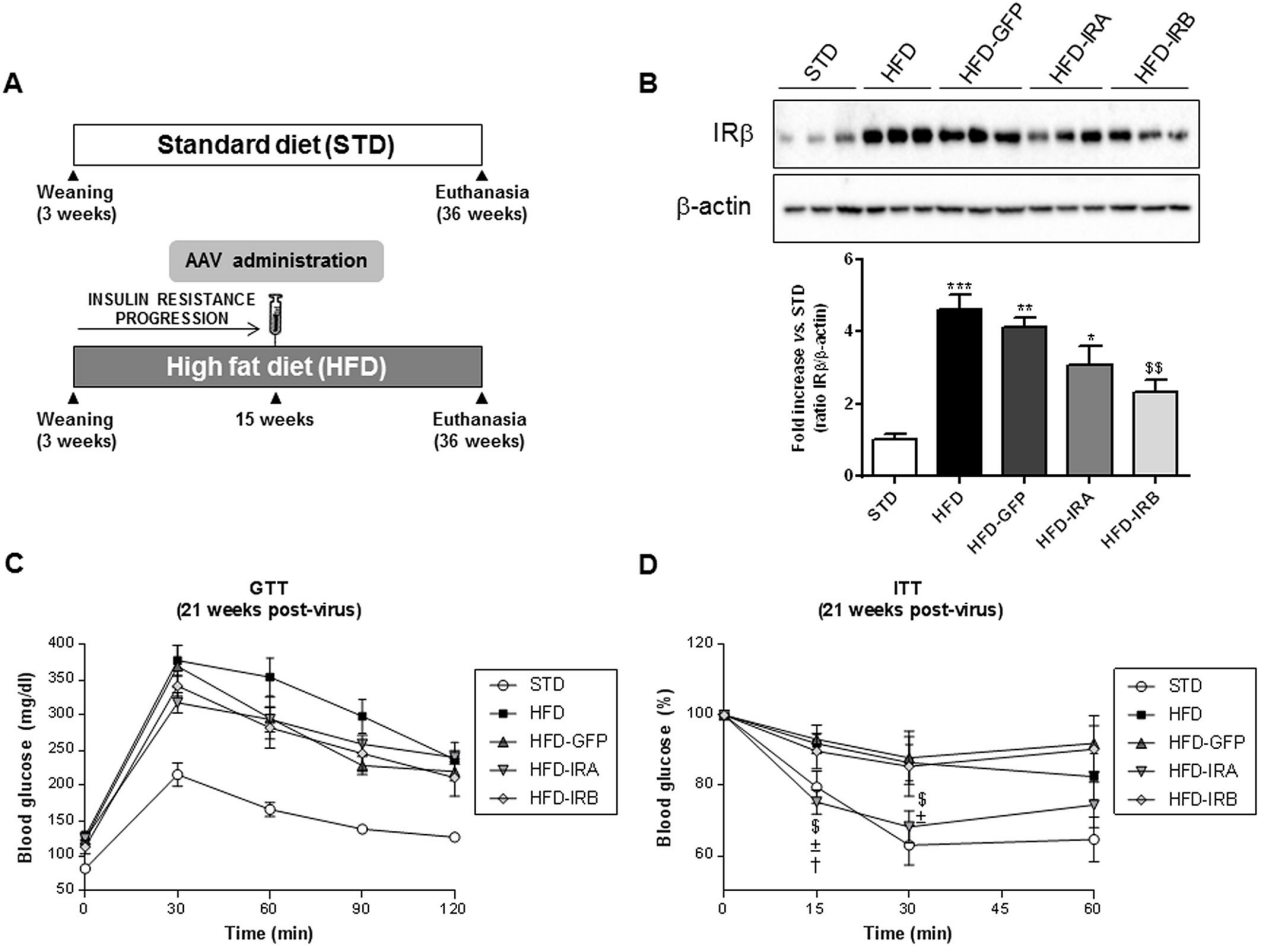
**Hepatic IRA expression ameliorates insulin tolerance.** (A) Scheme of diet and AAV administration. The mouse groups were established as follows: STD (*n*=5), HFD (*n*=9), HFD-GFP (*n*=6), HFD-IRA (*n*=8) and HFD-IRB (*n*=7). (B) Western blot analysis of IR in liver homogenates from the five groups studied (top). β-actin was used as a loading control. Histogram showing the IRβ/β-actin ratio quantification of band intensities (bottom). (C) GTT in STD (*n*=5), HFD (*n*=9), HFD-GFP (*n*=9), HFD-IRA (*n*=9) and HFD-IRB (*n*=9) animals at 21 weeks after AAV administration. (D) ITT in STD (*n*=5), HFD (*n*=9), HFD-GFP (*n*=9), HFD-IRA (*n*=9) and HFD-IRB (*n*=9) animals at 21 weeks after AAV administration. Results are expressed as mean±s.e.m. Statistical significance was assessed by one-way ANOVA with Bonferroni post test in B, and two-tailed unpaired Student's *t*-test in C and D [versus STD mice (*P<0.05, **P<0.01 and ***P<0.001), versus HFD mice (^$^*P*<0.05 and ^$$^*P*<0.01), versus HFD-GFP mice (^±^*P*<0.05) and versus HFD-IRB mice (^†^*P*<0.05)].

To evaluate the importance of hepatic IR isoforms in the regulation of global glucose homeostasis, we performed the experiments indicated in Fig. 2A. After weaning, animals were fed a STD or HFD. Fifteen weeks after HFD administration, when insulin resistance was developed, mice were injected with the corresponding AAVs. AAV serotype 8 (AAV8) containing human IR isoforms *INSR A*, *INSR B* or green fluorescent protein (GFP) were used. These genes were under the control of a hepatospecific promoter, α1-antitrypsin (AAT). In addition, the constructs were composed by polyadenylation signals (polyA tail) and were flanked by inverted terminal repeats (ITRs) of serotype 2. Therefore, those animals fed a HFD were divided into four groups: HFD, HFD-GFP, HFD-IRA and HFD-IRB.

After sacrifice, liver, pancreas and kidney were removed and analyzed by reverse transcription polymerase chain reaction (RT-PCR) to check the expression of IR isoforms (Fig. S1). In Fig. S1A (top) we performed RT-PCR with primers for the detection of mouse IR isoforms in the liver; as expected, all the experimental groups expressed both mouse IR isoforms but mainly IRB. Subsequently, we performed RT-PCR with primers for the detection of human IR isoforms in order to ensure that the different AAVs had worked properly within this organ. As shown in Fig. S1A (middle and bottom), the specific human IR isoforms were expressed in the corresponding experimental groups. In Fig. S1B and C, we performed RT-PCR with primers for the detection of human IR isoforms in pancreas and kidney, respectively. In both cases, as expected, there was no expression of human IR isoforms, except in hepatic samples of HFD-IRA and HFD-IRB mice used as positive controls. These results demonstrate that our gene therapy approach with AAV8 together with the AAT promoter ensures a specific hepatic expression of both IR isoforms.

Moreover, we checked, by western blotting, the expression of IR within the liver, and, as Fig. 2B shows, the HFD-induced insulin resistance triggers a significant increase in IR expression as a compensatory mechanism. In addition, AAV-mediated expression of IRA or IRB did not increase global IR expression compared with the HFD group, and, in HFD-IRB mice, induced a significant decrease in global IR expression, suggesting some kind of regulatory mechanism.

Twenty-one weeks after AAV administration, we performed GTTs and ITTs to analyze the phenotype reversion in terms of glucose homeostasis (Fig. 2C,D). Although we did not find any change in GTT results among the HFD-fed animals, it is noteworthy to emphasize that only HFD-IRA mice showed a significant amelioration in insulin tolerance, despite the fact that the animals were fed the HFD until euthanasia.

**Fig. 3.**
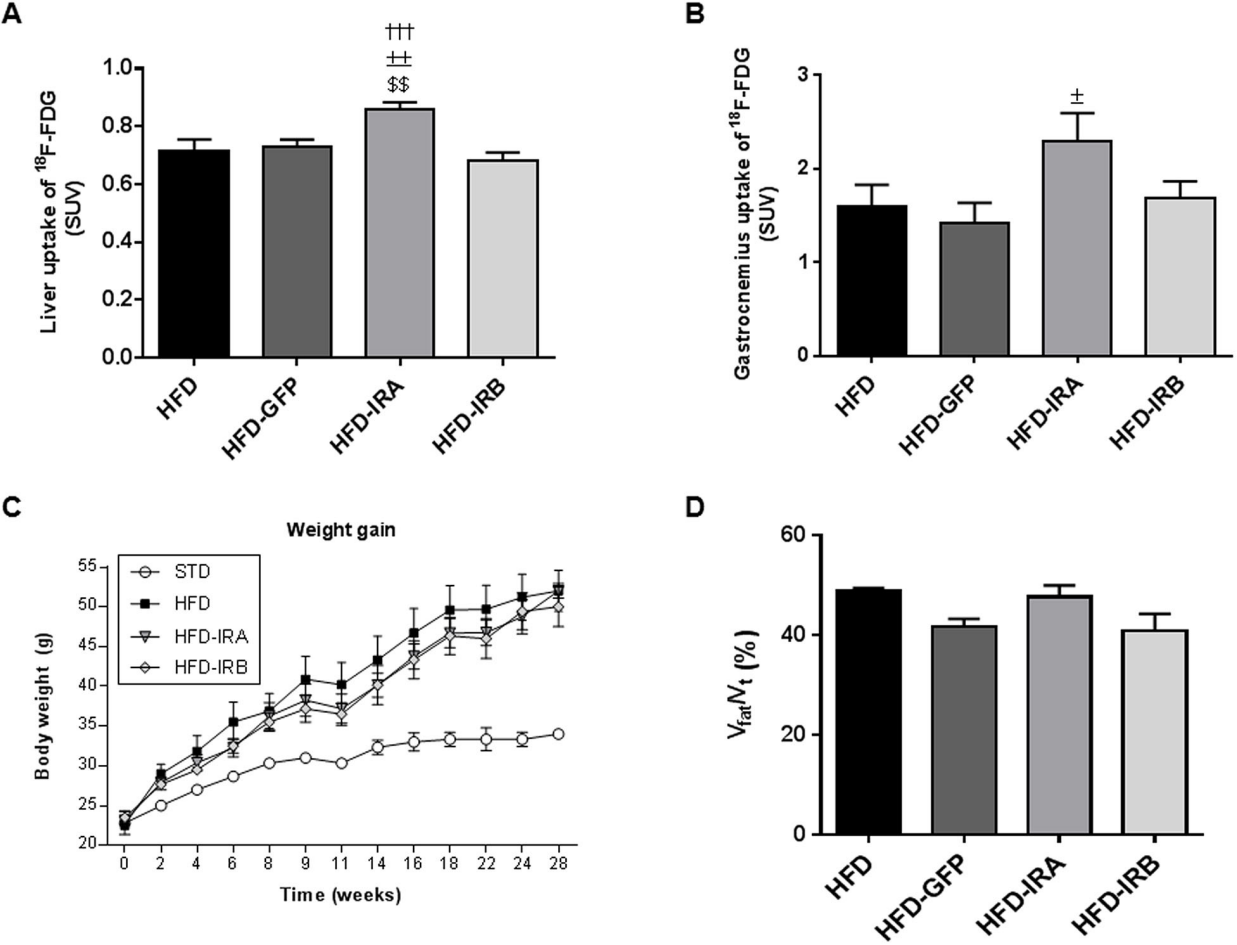
**AAV-mediated hepatic IRA expression induces an improvement in glucose uptake in liver and muscle.** (A) Quantification of *in vivo* hepatic glucose uptake represented as standard uptake values (SUVs). (B) Quantification of *in vivo* glucose uptake by gastrocnemius represented as SUVs. Animals analyzed by PET belonged to HFD (*n*=9), HFD-GFP (*n*=6), HFD-IRA (*n*=8) and HFD-IRB (*n*=7) groups. (C) Graph showing body weight gain in STD (*n*=5), HFD (*n*=9), HFD-IRA (*n*=8) and HFD-IRB (*n*=7) mice from weaning to 28 weeks of age. (D) Quantification of NMR images after AAV injection of mice in HFD (*n*=8), HFD-GFP (*n*=9), HFD-IRA (*n*=9) and HFD-IRB (*n*=9) groups. Results are expressed as mean±s.e.m. Statistical significance was assessed by two-tailed unpaired Student's *t*-test [versus HFD mice (^$$^*P*<0.01), versus HFD-GFP mice (^±^*P*<0.05 and ^±±^*P*<0.01) and versus HFD-IRB mice (^†††^*P*<0.001)].

To further characterize the mechanisms involved in this improvement we performed positron-emission tomography (PET), and, as Fig. 3A shows, the HFD-fed mice with hepatic expression of IRA exhibited a significant increase in [^18^F]-2-fluoro-D-2-deoxy-D-glucose (^18^F-FDG) uptake by the liver compared with the other groups studied. We also wanted to establish whether this increase in liver glucose uptake could ameliorate the glucose uptake in muscle, and our results show that the gastrocnemius glucose uptake in HFD-IRA mice is higher than that in the other groups, although the difference was only statistically significant when compared with the HFD-GFP group, suggesting that hepatic IRA expression induces a global glucose uptake improvement (Fig. 3B). However, we did not find any change in weight gain or fat accumulation among the groups of mice fed a HFD (Fig. 3C,D).

**Fig. 4.**
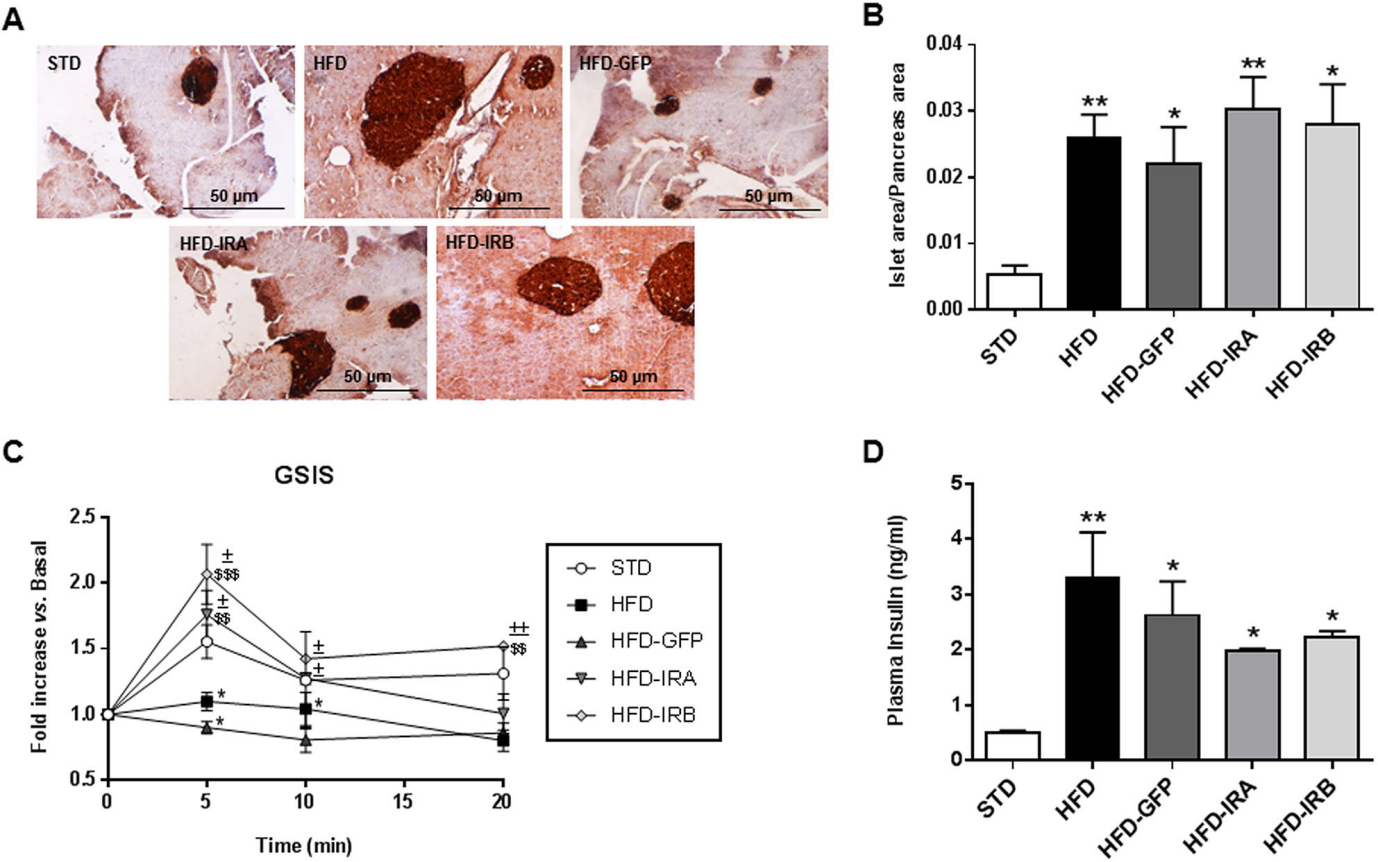
**Hepatic IR isoform expression improves first-phase insulin secretion.** (A) Representative images of pancreas immunohistochemistry with anti-insulin labeling. Image magnification: ×10. (B) Quantification of islet area/pancreas area ratio of each experimental group (*n*=5). (C) Glucose-stimulated insulin secretion (GSIS) *in vivo* in all groups studied (*n*=6). (D) Quantification of plasma insulin levels (ng/ml) in 16 h-fasted animals by ELISA (*n*=6). Results are expressed as mean±s.e.m. Statistical significance was assessed by two-tailed unpaired Student's *t*-test [versus STD mice (*P<0.05 and **P<0.01), versus HFD mice (^$$^*P*<0.01 and ^$$$^*P*<0.001) and versus HFD-GFP mice (^±^*P*<0.05 and ^±±^*P*<0.01)].

To study the compensatory mechanisms associated with insulin resistance and the impact of the hepatic expression of IR isoforms on this response, we performed immunohistochemical analysis of pancreatic sections from all the groups studied by staining with anti-insulin antibodies (Fig. 4A). The quantification of islet area/pancreas area ratio showed a higher value for all the groups under a HFD; however, hepatic IR expression did not affect this parameter in any case (Fig. 4B). Moreover, 2 weeks before sacrifice we performed glucose-stimulated insulin secretion (GSIS) assays. The results revealed that hepatic gene therapy with human IR isoforms enables the recovery of first-phase insulin secretion, lost in HFD and HFD-GFP mice (Fig. 4C). We also analyzed plasma insulin levels and found that all HFD-fed mice showed a significant increase compared with the STD group. That increase was less dramatic in HFD-IRA or HFD-IRB mice, showing a trend towards reducing systemic insulin levels (Fig. 4D).

**Fig. 5.**
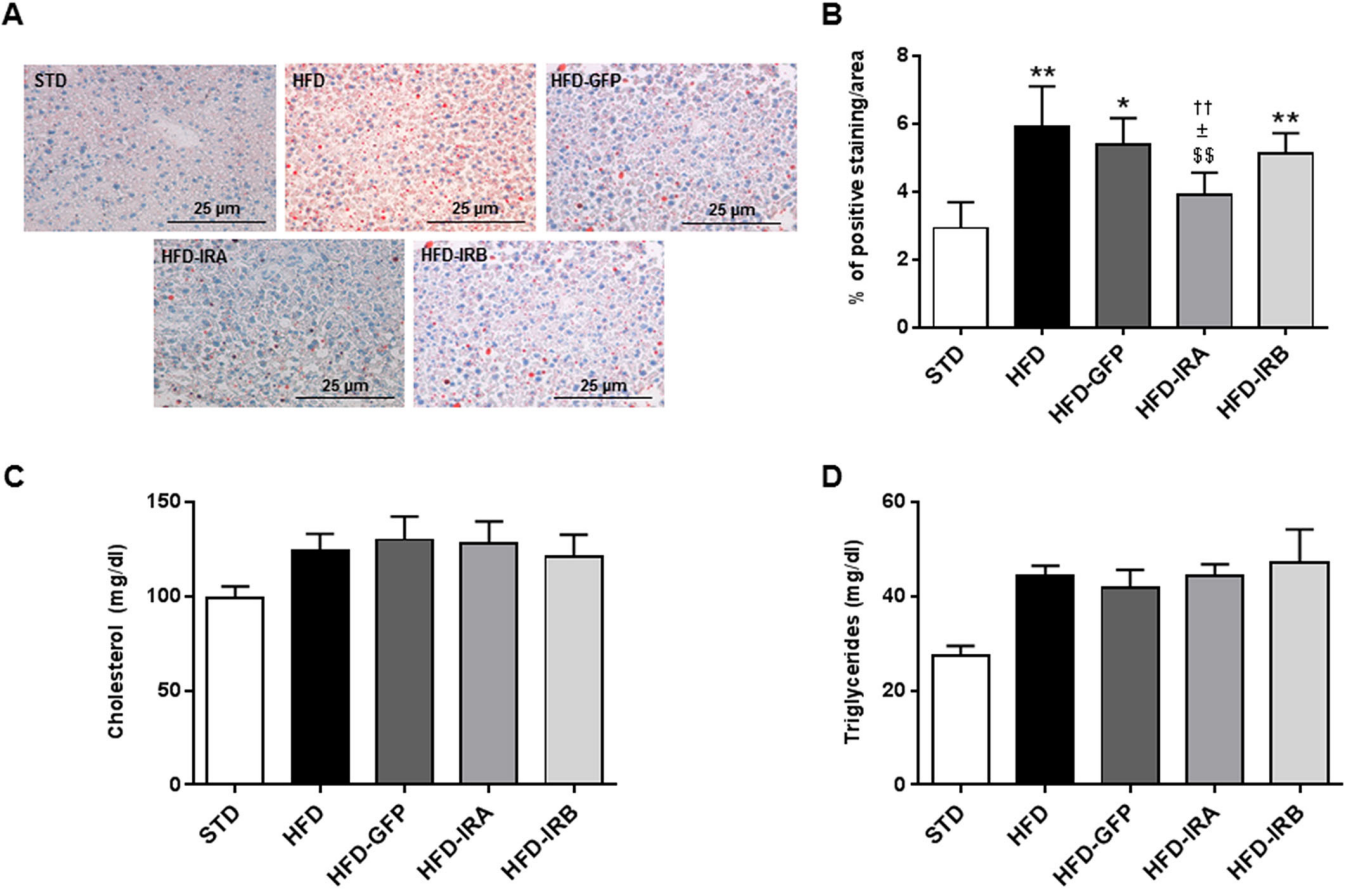
**Hepatic IRA expression alleviates** **intrahepatic lipid accumulation.** (A) Representative images of Oil Red O staining to study hepatic-specific lipid accumulation. Image magnification: ×20. (B) Quantification of hepatic-specific lipid content in STD (*n*=5), HFD (*n*=6), HFD-GFP (*n*=5), HFD-IRA (*n*=6) and HFD-IRB (*n*=6) mice. (C) Circulating cholesterol levels (mg/dl) in STD (*n*=5), HFD (*n*=8), HFD-GFP (*n*=6), HFD-IRA (*n*=8) and HFD-IRB (*n*=6) mice. (D) Circulating triglyceride levels (mg/dl) in STD (*n*=5), HFD (*n*=8), HFD-GFP (*n*=6), HFD-IRA (*n*=8) and HFD-IRB (*n*=7) mice. Results are expressed as mean± s.e.m. Statistical significance was assessed by two-tailed unpaired Student's *t*-test [versus STD mice (*P<0.05 and **P<0.01), versus HFD mice (^$$^*P*<0.01), versus HFD-GFP mice (^±^*P*<0.05) and versus HFD-IRB mice (^††^*P*<0.01)].

Given that NAFLD is one of the main complications associated with obesity and insulin resistance, our next step was to investigate whether hepatic IR isoform expression could affect the lipid accumulation within this organ. For this reason, we performed Oil Red O stains in liver sections from all the groups studied. In HFD mice, we observed a significant increase in the percentage of Oil Red O-positive cells compared with STD-fed mice; the same was observed for HFD-GFP animals, but, in the case of HFD-IRA mice, this increase was not observed. In fact, we observed a significant decrease in liver lipid accumulation compared with the HFD, HFD-GFP and HFD-IRB mice. The results obtained for the HFD-IRB mice were very similar to those obtained for the HFD and HFD-GFP mice (Fig. 5A,B). Although we observed an improvement in fat accumulation in HFD-IRA mice, this was not correlated with significant changes in circulating levels of cholesterol and/or triglycerides (Fig. 5C,D).

**Fig. 6.**
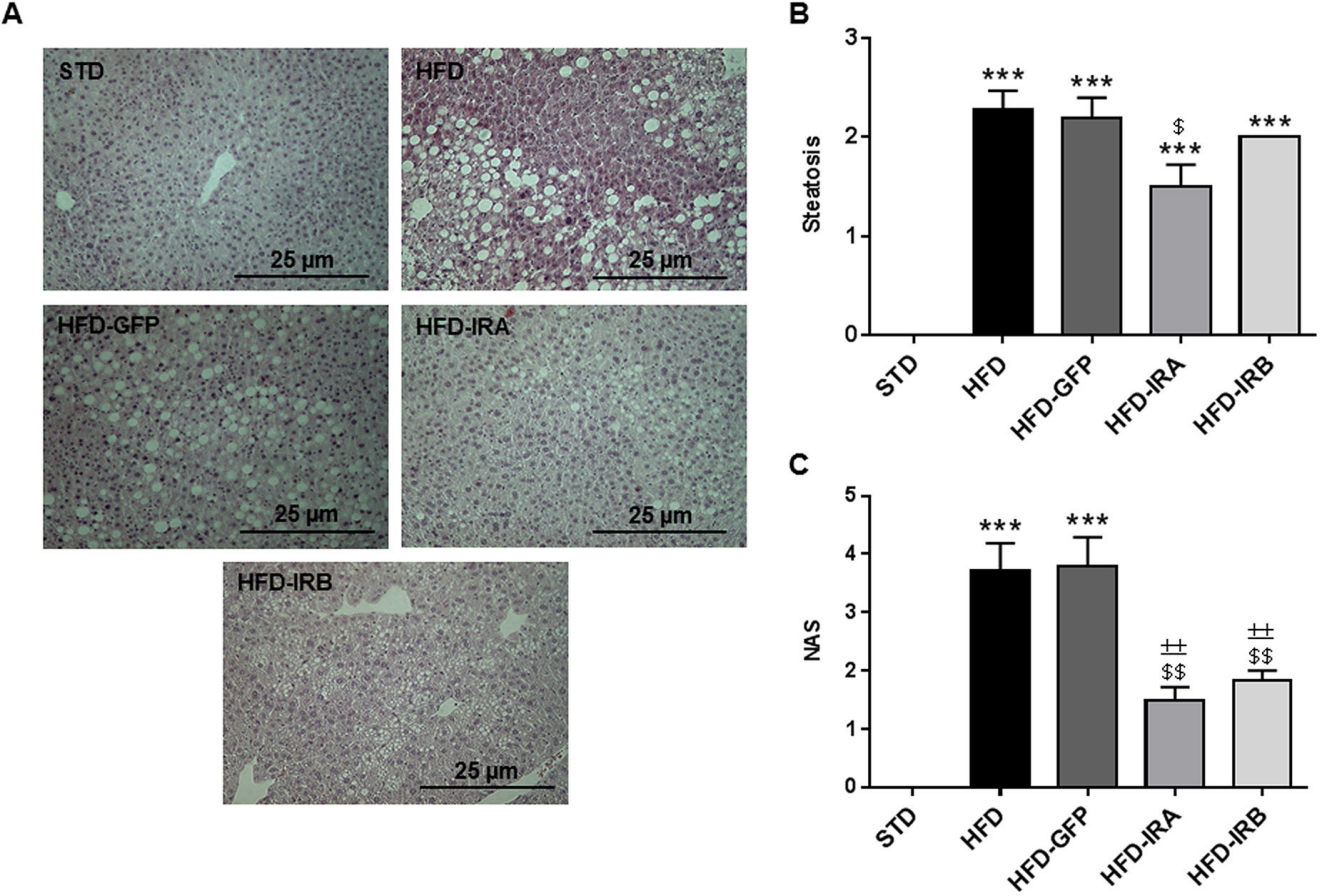
**IRA and IRB expression in the liver improves the NAFLD degree.** (A) Representative images of liver histological analysis through H&E staining. Image magnification: ×20. (B) Quantification of steatosis degree (*n*=7 per group). (C) Quantification of NAS according to liver abnormalities in all groups (*n*=7 per group). Results are expressed as mean±s.e.m. Statistical significance was assessed by one-way ANOVA with Bonferroni post test [versus STD mice (***P<0.001), versus HFD mice (^$^*P*<0.05 and ^$$^*P*<0.01) and versus HFD-GFP mice (^±±^*P*<0.01)].

To further explore the hepatic phenotype upon gene therapy, we performed Hematoxylin and Eosin (H&E) staining (Fig. 6A), evaluated by a pathologist specializing in hepatic histopathology from Santa Cristina Hospital (Madrid, Spain). The first conclusion from these experiments was that AAV-mediated hepatic IR expression does not induce any structural alteration in the liver. Moreover, the four groups of HFD-fed mice developed a significant degree of steatosis compared with STD-fed mice. However, only HFD-IRA mice showed significant regression compared with the HFD group (Fig. 6B). These data suggest that the expression of IRA could be partially protecting from excessive lipid accumulation and the development of NAFLD and subsequent complications such as cirrhosis. We also analyzed the NAFLD activity score (NAS) and found a significant increase in NAS in HFD and HFD-GFP mice compared with STD mice, whereas the expression of IRA or IRB caused a significant improvement in this index, suggesting that the expression of both isoforms improved the obesity-associated hepatic steatosis (Fig. 6C).

**Fig. 7.**
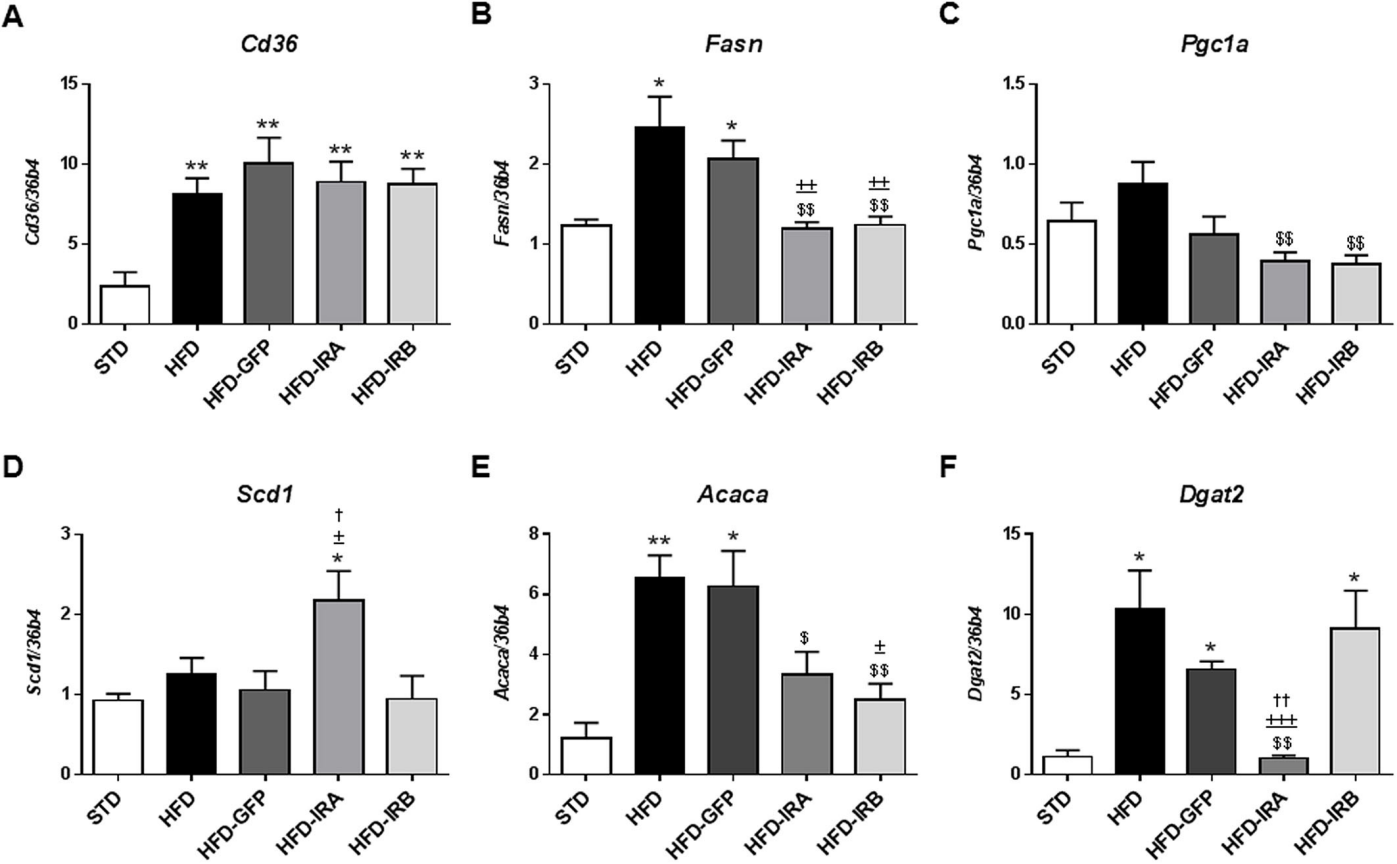
**Hepatic IRA expression alters mRNA levels of key genes involved in hepatic lipid metabolism.** mRNA levels of *Cd36*, *Fasn*, *Pgc1a*, *Scd1*, *Acaca* and *Dgat2* by quantitative RT-PCR in STD (*n*=5), HFD (*n*=9), HFD-GFP (*n*=6), HFD-IRA (*n*=8) and HFD-IRB (*n*=7) mice. *36b4* was used as a control. Results are expressed as mean±s.e.m. Statistical significance was assessed by two-tailed unpaired Student's *t*-test [versus STD mice (*P<0.05 and **P<0.01), versus HFD mice (^$^*P*<0.05 and ^$$^*P*<0.01), versus HFD-GFP mice (^±^*P*<0.05, ^±±^*P*<0.01 and ^±±±^*P*<0.001) and versus HFD-IRB mice (^†^*P*<0.05 and ^††^*P*<0.01)].

Next, we evaluated the mRNA levels of key genes involved in hepatic lipid metabolism (Fig. 7). Our results show that *Cd36* mRNA levels are elevated in all HFD-fed groups compared with STD mice, but no changes were induced by IR isoform expression (Fig. 7A). Fatty acid synthase (*Fasn*), peroxisome proliferator-activated receptor-gamma coactivator (PGC)-1 alpha (*Pgc1a*; also known as *Ppargc1a*) and acetyl-CoA carboxylase alpha (*Acaca*) mRNA levels were significantly increased in HFD mice, but IRA and IRB expression reverted this increase (Fig. 7B,C,E). Moreover, we studied the mRNA levels of stearoyl CoA desaturase 1 (*Scd1*) and found that only HFD-IRA mice showed a significant increase compared with the other groups assayed (Fig. 7D). Finally, mRNA levels of diacylglycerol O-acyltransferase 2 (*Dgat2*) were significantly elevated in HFD, HFD-GFP and HFD-IRB mice, but not in HFD-IRA mice (Fig. 7F).

## DISCUSSION

The current global epidemic of obesity has led to a huge increase in the metabolic diseases associated with this condition, which has focused the attention on the mechanisms involved in this disease and its comorbidities. Many of those, including T2DM, CVDs, NAFLD and even neurodegenerative diseases, are related to low-grade chronic inflammation (Hotamisligil, 2006; Lumeng and Saltiel, 2011; Olefsky and Glass, 2010). The causal relationship between inflammation and the complications of obesity remains obscure; however, it is widely accepted that obesity is closely associated with inflammation and that the degree of inflammation correlates well with the severity of insulin resistance and T2DM (Hotamisligil, 2006; Kotas and Medzhitov, 2015; Lumeng and Saltiel, 2011; Olefsky and Glass, 2010).

Excess calories are initially stored in subcutaneous fat; but, when this storage capacity is overwhelmed, the altered endocrine functions of adipose tissues and the ensuing ectopic fat accumulation lead to a lipotoxic metabolic stress, which promotes low-grade inflammation and metabolic dysfunction in organs such as liver or skeletal muscle, thereby promoting insulin resistance. The metabolic abnormalities associated with obesity predispose patients to cardiometabolic complications such as dyslipidemia, T2DM and NAFLD, which put them at risk of developing CVD (Van Gaal et al., 2006).

The liver has a major role in the regulation of glucose homeostasis by rapidly adapting to changes in blood glucose concentration (El Ouaamari et al., 2016). T2DM patients show unbalanced insulin signaling (Malaguarnera and Belfiore, 2011); indeed, in insulin-resistant states, insulin does not suppress hepatic glucose production but it retains its capacity to drive lipogenesis (Cook et al., 2015). This selective insulin resistance in T2DM is crucial for therapy. It could be preferable to develop new drugs able to improve insulin sensitivity, leading to the suppression of hepatic glucose output, as well as agents capable of enhancing glucose uptake in order to diminish the hyperglycemia (Brown and Goldstein, 2008). According to this, our group reported that, *in vitro*, the expression of IRA, but not IRB, improved glucose uptake in murine hepatocytes and beta cells by its direct association with GLUTs (Escribano et al., 2015; Nevado et al., 2006). Taking into account that glucose uptake is insulin independent in the liver, these changes cannot be related to insulin binding. Therefore, it is necessary to dissect the *in vivo* role of each isoform in the liver in the context of T2DM. Recently, our group demonstrated that the differential expression of IR isoforms in the liver triggers different responses in terms of glucose homeostasis. Concisely, our results revealed that, in the murine model of hepatic insulin resistance, iLIRKO, the specific hepatic expression of IRA mediated by AAV was able to ameliorate glucose intolerance, to reduce hyperinsulinemia and even to induce a regression in beta-cell hyperplasia (Diaz-Castroverde et al., 2016b). Moreover, we also demonstrated that the AAV-mediated hepatic IRA expression was able to increase glycogen accumulation in the liver of iLIRKO mice, suggesting that the increased glucose uptake induced by IRA expression is followed by glycogen synthesis (Diaz-Castroverde et al., 2016a). With this background, we hypothesized that hepatic IRA expression could serve as a glucose uptake facilitator, improving hyperglycemia, one of the main characteristics of insulin-resistant states. In the current study, the effects of AAV-mediated hepatic IR isoform expression were evaluated in a mouse model of diet-induced insulin resistance. Here, we report that, *in vivo*, HFD-fed mice with hepatic expression of IRA exhibit significantly increased liver uptake of ^18^F-FDG compared with the other groups studied, probably due to the direct association of IRA with GLUT-1 and GLUT-2, as previously reported in murine hepatocytes (Nevado et al., 2006) and beta cells (Escribano et al., 2015). In the same way, the gastrocnemius uptake of ^18^F-FDG was significantly enhanced in HFD-IRA versus HFD-GFP mice. These data could suggest that the improvement in hepatic glucose uptake leads to a global increase in insulin tolerance, as observed in the ITTs, which positively affects the muscular glucose uptake. Beta-cell mass increases to compensate for obesity-associated insulin resistance, causing hyperinsulinemia (Cook et al., 2015; Escribano et al., 2009; Lingohr et al., 2002; Michael et al., 2000; Stumvoll et al., 2005). Thus, although all HFD-fed groups showed an increased islet area/pancreas area ratio, only the mice with hepatic expression of IR isoforms showed a trend towards reduced systemic insulin levels and a recovery of the first phase of insulin secretion, demonstrating that AAV-mediated specific hepatic expression of IR isoforms regulates the compensatory mechanisms of insulin resistance.

NAFLD is one of the main comorbidities of obesity (Targher et al., 2018) and has become one of the most common chronic liver diseases around the world; its prevalence is even greater in T2DM patients (Anstee et al., 2013; European Association for the Study of the Liver et al., 2016; Italian Association for the Study of the Liver, 2017). Moreover, patients with T2DM and NAFLD are more likely than patients with NAFLD alone to develop the more severe forms of NAFLD [that is, non-alcoholic steatohepatitis (NASH), advanced fibrosis and cirrhosis], which can ultimately lead to hepatocellular carcinoma (Anstee et al., 2013; European Association for the Study of the Liver et al., 2016; Italian Association for the Study of the Liver, 2017). With this background, we studied intrahepatic lipid accumulation in our experimental groups and wondered whether the hepatic expression of IR isoforms could alleviate this process. Our results demonstrate that the HFD-fed mice exhibited a significant increase in lipid accumulation compared with the STD-fed mice. However, only the AAV-mediated IRA-expressing group showed a significant reduction in lipid accumulation, suggesting that the expression of IRA within the liver not only increases glucose uptake and ameliorates insulin sensitivity, but is also able to reduce hepatic lipid accumulation, alleviating one of the main pathologies associated with obesity. To further characterize the effect of hepatic IR isoform expression in obesity-related liver damage, liver sections were stained with H&E and evaluated by an expert pathologist in hepatic histopathology. The results showed that all the groups under a HFD developed a significant degree of steatosis, with the IRA-expressing group the least affected, reinforcing the data obtained from the Oil Red O stains. In addition, the NAS was significantly higher in the HFD and HFD-GFP groups than in STD mice, although both the HFD-IRA and HFD-IRB groups did not show any significant change compared with STD mice, demonstrating that AAV-mediated hepatic expression of both IR isoforms does not cause any hepatic alteration and also protects from obesity-induced liver damage. In this sense, the main changes observed in NAS between HFD and HFD-GFP versus HFD-IRA and/or HFD-IRB groups were related to inflammation foci and ballooning. The mice that received AAVs containing IRA or IRB did not show any inflammation foci or ballooning within the liver, whereas HFD and HFD-GFP mice showed both. Thus, according to our results, one possible explanation is that the hepatic expression of IR isoforms alters the gene expression of key enzymes related to fatty acid synthesis, alleviating the toxicity of free fatty acids and the subsequent inflammation.

To investigate the underlying molecular mechanisms involved, we evaluated mRNA levels of key proteins in hepatic lipid metabolism. First, we analyzed the mRNA levels of the non-esterified fatty acid transporter CD36. Our results showed that *Cd36* mRNA levels are significantly increased in the liver of all HFD-fed groups, as previously described in mouse models and in patients with steatosis (Buque et al., 2010; Degrace et al., 2006; Inoue et al., 2005; Miquilena-Colina et al., 2011). Next, we studied the mRNA levels of *Fasn*. Our results showed that this enzyme increased in HFD mice, as previously described in the liver of NAFLD models (Kohjima et al., 2007). However, the hepatic expression of IRA or IRB by AAV induced a significant decrease in the levels of *Fasn*, suggesting that the ectopic expression of IR isoforms could alleviate the increased palmitic acid synthesis that aggravates NAFLD. However, although this mechanism could be involved in the decreased NAS observed in HFD-IRA and HFD-IRB mice, the hepatic steatosis did not improve in the liver of HFD-IRB mice, suggesting that the above mechanism is necessary, but not sufficient, to ameliorate liver steatosis.

Moreover, we studied the mRNA levels of *Pgc1a*, a transcription factor that stimulates gluconeogenesis by inducing the expression of phosphoenolpyruvate carboxykinase (PEPCK; also known as Pck1) and glucose-6-phosphatase (G6Pase; also known as G6pc) (Yoon et al., 2001). Our results revealed that the hepatic expression of IRA and IRB induced a significant decrease in mRNA levels of *Pgc1a*, demonstrating that this approach is able to inhibit gluconeogenic genes, alleviating hyperglycemia. Furthermore, we analyzed *Scd1* mRNA levels; our results demonstrate that only in livers from HFD-IRA mice was there a significant increase in *Scd1* mRNA levels compared with the other groups studied. Given that SCD1 catalyzes the formation of monounsaturated fatty acids (MUFAs) from saturated fatty acids (SFAs), and that an increase in the MUFA/SFA ratio has been correlated with an improvement in both glucose and lipid metabolism (Benhamed et al., 2012), the increase in *Scd1* mRNA levels observed in HFD-IRA mice, together with the decrease in *Fasn* and *Pgc1a* mRNA levels, could explain the improvement in glucose homeostasis and liver steatosis observed in HFD-IRA mice.

Regarding *Acaca* mRNA levels, the data obtained revealed an increase in HFD and HFD-GFP groups, whereas the hepatic expression of IRA or IRB by AAV induced a significant decrease. This enzyme also has a crucial role in fatty acid synthesis and it has been previously described that decreased hepatic expression of *Acaca* improves fatty liver profile (Samuel and Shulman, 2018). Finally, we analyzed the mRNA levels of the acetyltransferase *Dgat2*. Given that high levels of this enzyme are related to enhanced lipid accumulation (Virtue and Vidal-Puig, 2010), the decrease observed in HFD-IRA mice might explain the improvement in steatosis.

In summary, our results demonstrate that AAV-mediated IRA expression in the liver of HFD-fed mice increased hepatic glucose uptake and decreased lipid accumulation, reducing – or at least delaying – the development of fatty liver and NASH. It is noteworthy to emphasize that IRA expression in the liver did not cause any structural dysplasia even after 21 weeks of viral administration. Therefore, we can conclude that IRA expression in the liver could be a safe gene therapy approach to improve hepatic glucose consumption and liver steatosis associated with obesity.

## MATERIALS AND METHODS

### Mice and diets

Male mice from the C57BL/6 strain and wild-type genotype were purchased from the Jackson Laboratory and maintained on a 12 h light/dark cycle. Animals were fed *ad libitum* from weaning until sacrifice, and five groups of seven to nine mice were established. After weaning, one group of mice was fed a STD (18% calories from fat; TD 2014, Envigo Teklad, Barcelona, Spain). The other mice were fed a HFD (60% calories from fat; TD 06414, Envigo Teklad) and were sorted into four groups: (1) HFD (only HFD); (2) HFD-GFP (HFD chow and AAV-GFP administration); (3) HFD-IRA (HFD chow and AAV-IRA administration); and (4) HFD-IRB (HFD chow and AAV-IRB administration). All animal experimentation was conducted in accordance with the accepted standards of animal use approved by the Complutense University of Madrid Committee.

### Viral constructs and vector production and purification

Recombinant AAV vectors were constructed with a transgene cassette coding sequence for the individual spliced single-chain isoforms of *INSR* either containing or lacking exon 11 (IRB and IRA, respectively). The viral particles were obtained from HEK293T packaging cells. Recombinant vectors carry AAV8 and contain the human IR transgene, either IRA or IRB (GenBank accession number NM_001079817.2 and NM_000208.3, respectively), under the regulation of a murine liver-specific promoter, AAT (Kattenhorn et al., 2016; Vanrell et al., 2011), including an adenine tail [poly(A)]. Coding sequences for the human IR isoforms were a generous gift from C. R. Kahn (Joslin Diabetes Center, Boston, MA, USA). The transgene cassette was flanked by AAV2 wild-type ITRs. rAAV8 vectors were produced as previously described (Gil-Farina et al., 2013). As a control, *INSR* was replaced by GFP (GenBank accession number L29345) to generate AAV-GFP.

### AAV administration

AAVs were administered by intravenous (i.v.) injection to 4-month-old mice. For all procedures, animals were anesthetized by intraperitoneal (i.p.) injection of a mixture of xylacine [Rompun 2% (wt/vol), Bayer, Leverkusen, Germany] and ketamine (Imalgene 50, Merial, Lyon, France) at 1:9 vol/vol. Four months post-AAV administration, the mice were euthanized for further analysis.

### Metabolic tests

GTTs were performed by i.p. glucose administration (2 g/kg body weight) in overnight (16 h)-fasted mice. The measurements were performed in a glucometer every 30 min using Accu-Check^®^ Aviva blood glucose strips (Roche, Penzberg, Germany). ITTs were performed in the random-fed state and the animals were injected with 1 U/kg body weight of human regular insulin (Humulin regular, Eli Lilly, Indianapolis, IN, USA) and blood glucose levels were measured at indicated times. ITT data are presented as percentage of initial blood glucose concentration. Acute insulin release experiments were performed 2 weeks prior to sacrifice as previously described (Gomez-Hernandez et al., 2012).

Insulin enzyme-linked immunosorbent assay (ELISA; Millipore, Billerica, MA) and assay of total cholesterol and total triglyceride (Thermo Fisher Scientific, Waltham, MA, USA) were performed with plasma samples obtained from overnight (16 h)-fasted mice.

### RT-PCR

RNA from liver, pancreas and kidney was prepared using Trizol^®^ (Thermo Fisher Scientific, Carlsbad, CA, USA) as described (Chomczynski and Sacchi, 2006), complementary DNA (cDNA) was synthesized using a High-Capacity cDNA Reverse Transcription kit (Thermo Fisher Scientific) and PCR was performed with DNA AmpliGel Master Mix (Biotools, Madrid, Spain). Primers flanking the mouse exon 11 were 5′-ATCAGAGTGAGTATGACGACTCGG-3′ and 5′-TCCTGACTTGTGGGCACAATGGTA-3′. Primers flanking the human exon 11 were 5′-AGGAAGACGTTTGAGGATT-3′ and 5′-CACCGTCACATTCCCAACAT-3′.

Analysis of gene expression (*Cd36*, *Fasn*, *Pgc1a*, *Scd1*, *Acaca*, *Dgat2*) was performed by quantitative RT-PCR in an ABI Prism 7900HT Thermal Cycler (Thermo Fisher Scientific) using TaqMan^®^ Universal PCR Master Mix (Thermo Fisher Scientific) and TaqMan probes for the corresponding genes. The relative abundance of mRNAs was calculated using *36b4* (also known as *Rplp0*) mRNA as a reference. The results were calculated using the 2^-ΔΔCq^ method.

### Western blotting

Western blot analyses were performed on liver homogenates as previously described (Escribano et al., 2003). The antibodies used were anti-IRβ (sc-711; 1:2000 in TTBS) from Santa Cruz Biotechnology (Dallas, TX, USA) and anti-β-actin (A2228; 1:5000 in TTBS) from Sigma-Aldrich (St. Louis, MO, USA). Rabbit and mouse primary antibodies were immunodetected using horseradish peroxidase-conjugated anti-rabbit (NA931V; 1:4000 in TTBS) or anti-mouse (NA934V; 1:5000 in TTBS) (GE Healthcare, Buckinghamshire, UK) antibody, respectively. Loading was normalized by β-actin. The band intensities were quantified using ImageJ v1.6 software (http://rsb.info.nih.gov/ij).

### PET/computed tomography analysis

In order to measure hepatic and muscular glucose uptake in mice fasted for 6-8 h, animals were anesthetized with isoflurane and injected (i.v.) with 140-150 μCi ^18^F-FDG. The results were analyzed with PMOD v3.0 software (Pmod Technologies) and presented as the standardized uptake values (SUVs).

### NMR

Mice were anesthetized, respiration was continuously monitored and body fat was measured using a Bruker BioSpec 47/40 (Bruker, Ettlingen, Germany). The results were presented as fat body volume versus total body volume using ImageJ v1.6 software.

### H&E and Oil Red O stains

Liver samples were included in Tissue-Tek^®^ optimum cutting temperature (OCT) compound (Sakura Finetek, Alphen aan den Rijn, The Netherlands), and later in liquid nitrogen for freezing. Each liver block was serially sectioned (7 μm) with a cryostat (CM1510 S, Leica, Wetzlar, Germany) to perform the H&E and Oil Red O stains using standard techniques. All liver sections measured >1.5 cm in length and showed more than ten complete portal tracts. The percentage of hepatocytes containing lipid droplets was determined for each 10× field. An average percentage of steatosis was then determined for the entire specimen. Images of sections of both H&E and Oil Red O were acquired using an inverted Eclipse TE300 microscope (Nikon, Tokyo, Japan) coupled to a Digital Sight DS-U2 camera (Nikon). For images of Oil Red O stains, quantification was performed using IP Win32 v4.5 software (Acromag).

### Immunohistochemistry

Pancreas samples were fixed overnight in 4% formaldehyde made up in 10% phosphate-buffered saline (PBS) and routinely paraffin embedded. Each pancreas block was serially sectioned (7 μm) with a microtome (RM2125RT, Leica) to perform insulin immunohistochemistry. Pancreas sections were incubated with an antibody against insulin (ab7842, Abcam, Cambridge, UK) in 4% bovine serum albumin diluted in PBS 1× overnight at 4°C. The secondary antibody used was horseradish peroxidase-conjugated rabbit anti-guinea pig (1:200; ab6771) and, after that incubation, the development of immunocomplexes was carried out using a diaminobenzidine substrate kit for peroxidase (Dako, Glostrup, Denmark). Beta-cell fractional area was determined by calculating the ratio between the area occupied by insulin-positive cells and the area occupied by total pancreatic cells. Images of stained sections were acquired using an inverted Eclipse TE300 microscope (Nikon) coupled to a Digital Sight DS-U2 camera (Nikon), and the images were quantified using ImageJ v1.6 software.

### Diagnosis using NAS

In order to determine the degree of NAFLD, H&E staining was performed on paraffin-embedded liver sections (4 µm thick) that were evaluated by a single-blinded highly qualified hepatopathologist from Santa Cristina Hospital (Madrid, Spain). NAS was calculated for each liver biopsy studied as described (Kleiner et al., 2005). Briefly, the score is the sum of the scores for steatosis (0-3), lobular inﬂammation (0-3) and ballooning (0-2), so ranging from 0 to 8. Steatosis score was assessed, grading percentage involvement by steatotic hepatocytes as follows: grade 0, 0-5%; grade 1, >5-33%; grade 2, >33-66%; grade 3, >66%. In addition, Brunt's histological scoring system was used to evaluate the degree of hepatocellular ballooning and lobular inflammation (Brunt et al., 1999). The degree of lobular inflammation was measured, numbering the inflammatory foci per 200× field: grade 0, no foci; grade 1, <2 foci; grade 2, 2-4 foci; grade 3, >4 foci. The degree of hepatocellular ballooning was assessed according to the presence of balloon hepatocytes: grade 0, none; grade 1, few balloon cells; grade 2, many cells/prominent ballooning. Minimal criteria for steatohepatitis (NASH) included the combined presence of grade 1 steatosis, hepatocellular ballooning and lobular inflammation (Kleiner et al., 2005).

### Statistical analysis

Data are presented as mean±s.e.m. from at least four mice per group. Differences between two groups were assessed using unpaired two-tailed Student's *t*-tests. Data involving more than two groups were assessed by one-way analysis of variance (ANOVA) followed by Bonferroni tests, unless otherwise specified. *P*<0.05 was considered to be statistically significant. The software used for the analyses was GraphPad Prism v7.0 software.

## Supplementary Material

Supplementary information
