# Supplementary material for: Liver-specific insulin receptor isoform A expression enhances hepatic glucose uptake and ameliorates liver steatosis in a mouse model of diet-induced obesity
Source: Dis Model Mech. 2019 Feb 7;12(2):dmm036186. doi: 10.1242/dmm.036186 (PMC6398497; doi:10.1242/dmm.036186)
Supplement: Supplementary information [file dmm-12-036186-s1.pdf]

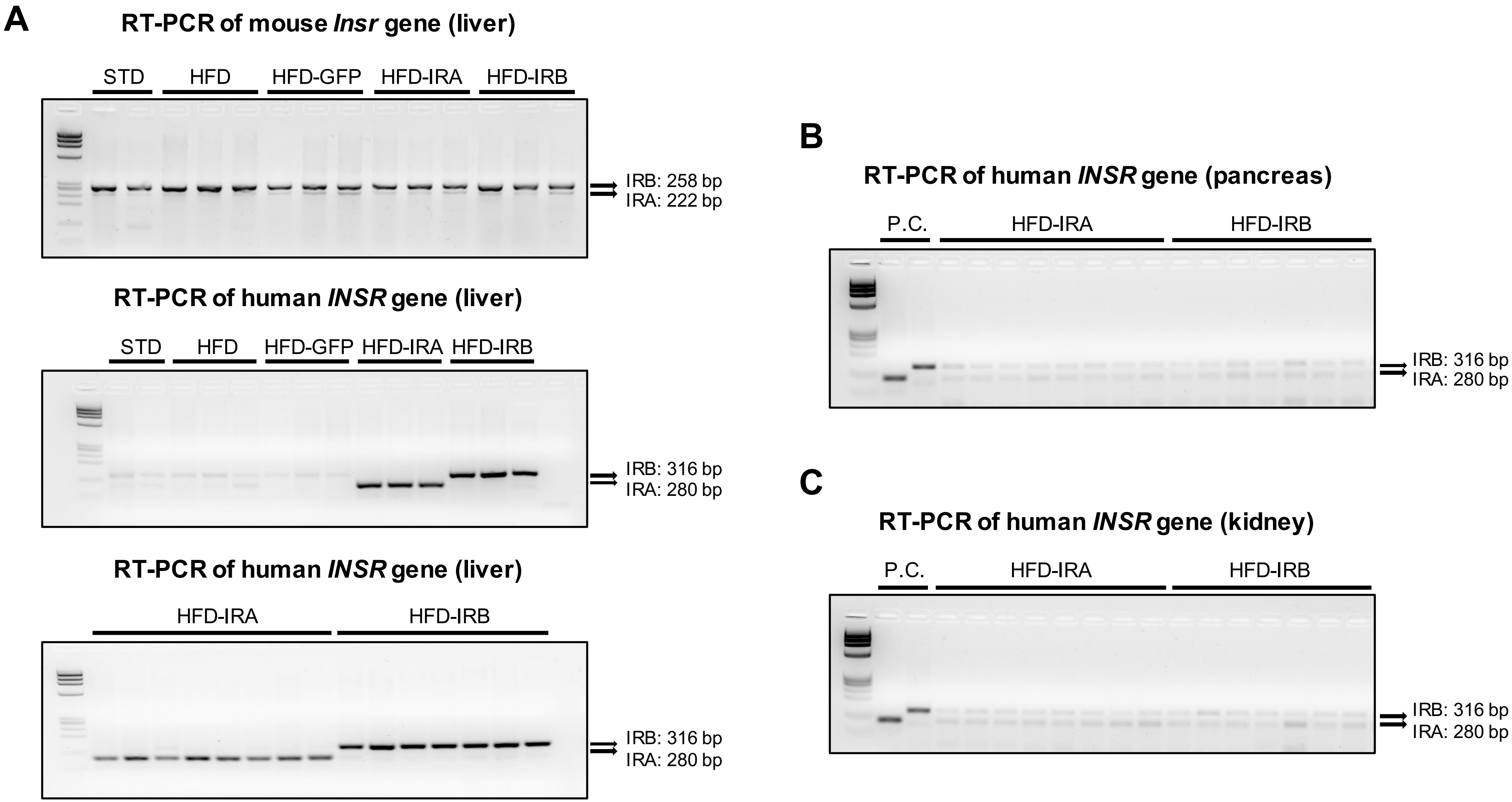

Fig. S1. Assessment that AAV-mediated IR isoforms expression is liver specific. A) RT-PCR analysis of IR isoforms expression in liver. *Upper panel*, RT-PCR of mouse *Insr* gene in STD (n=2); HFD (n=3); HFD-GFP (n=3); HFD-IRA (n=3) and HFD-IRB mice (n=3); *middle panel*, RT-PCR of human *INSR* gene in STD (n=2); HFD (n=3); HFD-GFP (n=3); HFD-IRA (n=3) and HFD-IRB mice (n=3); *lower panel*, RT-PCR of human *INSR* gene in HFD-IRA (n=8) and HFD-IRB mice (n=7). B) RT-PCR of human *INSR* gene in pancreas from HFD-IRA (n=8) and HFD-IRB mice (n=7). C) RT-PCR of human *INSR* gene in kidney from HFD-IRA (n=8) and HFD-IRB mice (n=7). Hepatic samples of HFD-IRA and HFD-IRB mice were used as positive controls (P.C.).
